# Supplementary material for: Learning curve and surgical outcome of robotic assisted colorectal surgery with ERAS program
Source: Sci Rep. 2022 Nov 29;12:20566. doi: 10.1038/s41598-022-24665-w (PMC9709162; doi:10.1038/s41598-022-24665-w)
Supplement: Supplementary file 2 — Supplementary Information 2. [file 41598_2022_24665_MOESM2_ESM.zip › RAL Raw data-Ñ[▒K/092/HS_ñw╝╨░O▒Kñσ.pdf]

正式摘要(電子簽章：000000219418377)		
W52 / W52 ☐ 英文標題(注意:無英文姓名-請補病人基本檔!)	☐【讀取】憑證	

臺 中 榮 民 總 醫 院

出院病歷摘要

轉入號 : 轉出號 :

[redacted]

**(8)**流水編號	
**(9)**入院日期	110/06/18 (2021)
**(10)**轉科日期	
**(11)**出院日期	110/06/23 (2021) 住院天數計 5 日

診斷

- 入院 :S-colon ca 15cm FAV
- 出院 :S-colon ca 15cm FAV s/p robotic assisted anterior resection on 2021/06/19

主訴

- bloody stool in 2021/05

病史

- This 35 y/o male without any systematic disease before suffered from bloody stool in 2021/05. He went to CRS OPD for help where colonscopy showed colon ca 15cm FAV and abdominal CT showed sigmoid colon tumor with metastatic lymphadenopathy which operation was suggested. This time, he was admitted for robotic assisted anterior resection.

體檢發現

-
  - General appearance :
    - easy looking , conscious clear , without cardiopulmonary distress
    - Vital signs自動帶入date 體溫(BT) 脈搏(P) 呼吸(R) 血壓(BP) 身高(BH) 體重(BW) BMI
      20210618 37.3℃ 77次/分 20次/分 147/97mmHg 173.1cm 87kg 29.0
    - Vital signs: B.P. 147/97 mmHg , P.R. 77 /min , R.R. 20 /min , B.T. 37.3 ℃
  - HEENT :
    - Eyes: no anemic conjunctiva, no icteric sclera, normal EOM
    - ENT: no otorrhea, no rhinorrhea, no erythematous mucosa, no injected tonsil, no deformity
  - Neck: supple, freely movable, trachea midline,no deformity, no engorged jugular vein , no palpable thyroid or lymph node enlargement
  - Chest:
    - Inspection: Grossly normal, symmetric and fully expansion, no accessory muscle used, no operation scar, no spider angiomata, no gynecomastia
    - Palpation: no palpable mass or nodule, no subcutaneous emphysema, no tenderness
    - Ausculation: clear breathing sound
  - Heart:
    - Percussion: normal heart size
    - Ausculation: regular heart beat, normal S1, S2, no gallop, no murmur
  - Abdomen:
    - Inspection: in
      - flat contour, symetrically, no scar, no stria, no dilated veins, no rashes
    - Ausculation: normoactive bowel sound, no abdominal bruits
    - palpation: soft, no muscle guarding or rigidity, no local tenderness, no rebounding pain, no palpable mass, no palpable pulsation
    - Percussion: no shifting dulllness, no hepatomegaly, no splenomagaly
-

Peripheral pulsation :	CA	BA	RA	FA	PA	PTA	DPA
Right side :	++	++	++	++	++	++	++/+++
Left side :	++	++	++	++	++	++	++/+++

  - Genito-anus: no tenderness or palpable mass lesion or rectal shelf, normal external genitalia, normal anal tone, brown stool

CRS CONDITION

-
  - Lung

手術日期及方法

- 手術日期及方法20210619 1. Robotic (XI) assisted low anterior resection
  2. Intraoperative colonoscopy : (-).
  3. High ligation (+) : At IMA root (Via robotic clips). IMV was ligated via endoclip.
  4. Take down from the splenic flexure : (-)
  5. Robotic EndoGIA (45-4.3) X2 + EEA (29mm) were applied
  6. Proximal cut end: > 8 cm, distal cut end: 5 cm
  7. No drainage tube was inserted
  8. ADHESION BARRIER FILM was placed below the central extended wound

住院治療經過

- After admission, pre op sruvey was OK and operation was don esmoothly. He could tolerated oral feeding gradually. Under relatively stable conditions, he will be discharged.

合併症

一般檢查

- 累積報告 :

DATE	TIME	ALB	TP	BIL.T	BIL.D	ALKP	AST	ALT	LDH	AMY	LIPASE	NH3	Ferritin	UA
1100619	1445	3.7		0.5			19	27						

DATE	TIME	WBC	RBC	HGB	HCT	MCV	PLT	BLST	NEUT	BAND	SEG	LYM	MON	EOS	BAS
1100618	1633	8070	4.39	12.9	37.9	86.3	367		67.4			24.5	5.7	1.9	0.5
1100619	1444	14990		13.4	40.0		408		84.4			13.8	1.5	0.0	0.3

DATE	TIME	NA	K	CL	BUN	CREAT	eGFR	CA	Mg	P	CRP	ESR	PCT	D-Dimer	LACTATE
1100619	1445	141	4.6		7	1.04		8.3							

特殊檢查

病理報告

其它

出院時情況

- 3.改門診治療

出院指示

藥名	劑量	劑量單位	用法	頻次	數量	數量單位	現況	天數	單位
$Biofermin tab	2	TAB	PO	TID	42	TAB	TH	7	天
(口)Takepron OD tab 30mg	1	TAB	PO	QDAC	7	TAB	TH	7	天
Ultracet tab	1	TAB	PO	QID	28	TAB	TH	7	天
Econ tab 40mg	1	TAB	PO	QID	28	TAB	TH	7	天

出院計劃

主治醫師_2062F 蔣鋒帆　/　住院醫師_2309E 古朋育

正式摘要(電子簽章：000000219418377)

W52 / W52

doctorRank:B
